# The Yin-Yang balance of SIRT1 and SIRT2 in cancer metabolic remodeling

**DOI:** 10.7150/ijbs.127696

**Published:** 2026-02-11

**Authors:** Fei Yi, Li Shen, Xindi Yang, Zhuo Wang, Xue Li, Zishi Shen, Wenting Liu, Qi Miao, Shuang Jiang, Eryan Kong, Xiaoyu Song, Tingting Zhou, Ning Bai, Liu Cao

**Affiliations:** 1 Health Sciences Institute, China Medical University, Shenyang, Liaoning 110122, China.; 2 Innovation Center of Aging Related Disease Diagnosis and Treatment and Prevention, Jinzhou Medical University, Jinzhou, Liaoning 121001, China.; 3 Institute of Psychiatry and Neuroscience, Henan Medical University, Xinxiang, Henan 453003, China.; 4 Clinical Translational Research Center, Shengjing Hospital of China Medical University, Shenyang, Liaoning 110002, China.

**Keywords:** SIRT1 and SIRT2, glucose metabolism, lipid metabolism, mitochondrial metabolism, tumor immune microenvironment

## Abstract

Sirtuin 1 (SIRT1) and Sirtuin 2 (SIRT2) are NAD⁺-dependent deacetylases that regulate cancer metabolic stress, exerting their effects primarily through post-translational modification of metabolic enzymes and transcription factors. They modulate glucose, lipid, and mitochondrial metabolism, as well as immune metabolism responses within the tumor microenvironment. Depending on cellular context, they can promote or suppress tumor growth by directing energy production, redox balance, and metabolic adaptation. These context-dependent and often opposing activities constitute a Yin-Yang mode of regulation in cancer metabolism, reflecting a dynamic balance between metabolic activation and constraint. Autophagy has emerged as a critical metabolic integration node regulated by both SIRT1 and SIRT2, linking nutrient sensing, mitochondrial quality control, and stress adaptation. This review summarizes recent advances in understanding how SIRT1 and SIRT2 coordinate tumor metabolism and discusses therapeutic strategies that target their regulatory balance to reprogram cancer metabolism. SIRT2 also functions as a metabolic checkpoint that restrains CD8⁺ T cell effector metabolism, providing a rationale for combining SIRT2 inhibition with immune checkpoint blockade in metabolically stressed tumor microenvironments.

## Introduction

Tumor development and progression are closely linked to metabolic disorders 1, 2. Cancer cells emerge within a metabolically demanding microenvironment marked by nutrient scarcity, hypoxia, and immune surveillance. To survive and proliferate, cancer cells reprogram key pathways in glucose, lipid, and mitochondrial metabolism and engage in crosstalk with immune cells. These adaptations are coordinated by signaling networks that couple metabolic state to transcriptional and post-translational regulation 3, 4.

Among the major regulators of these signaling networks are the Sirtuins, a family of NAD⁺-dependent deacetylases that link metabolic status to cellular responses. Sirtuin 1 (SIRT1) and Sirtuin 2 (SIRT2) are the most extensively studied and play critical roles in suppressing tumorigenesis 5, 6 and controlling tumor metabolism 7-9. Despite structural similarity, they play distinct regulatory roles. Whereas SIRT1 acts predominantly in the nucleus and mitochondria to control transcription factors and metabolic coactivators, SIRT2 functions mainly in the cytoplasm to regulate metabolic enzymes and signaling proteins 10-13.

The roles of SIRT1 and SIRT2 in cancer cannot be readily classified as simple tumor promoting or tumor suppressive categories 9, 14, 15. Their functions are highly context-dependent, varying with tumor type, microenvironmental conditions, and metabolic state. In certain contexts, SIRT1 promotes tumor growth by driving glucose and lipid metabolic reprogramming 16. In others, SIRT1 safeguards cells by maintaining genomic stability and stress responses 17, 18. Likewise, SIRT2 acts as a metabolic brake under physiological conditions, constraining excessive biosynthetic activity and maintaining cellular homeostasis. However, in pathological contexts SIRT2 has been linked to oncogenic processes, including stabilizing oncogenic proteins and supporting metabolic adaptations that promote tumor survival 19-21.

Recent studies suggest that the interplay between SIRT1 and SIRT2 exemplifies a Yin-Yang model of regulation. In cancer, this model reflects a dynamic balance in which SIRT1 and SIRT2 play opposing yet interdependent roles across glucose, lipid, mitochondrial, and immune metabolism 22, 23. Their relative contributions vary with metabolic state and microenvironmental stress, allowing coordinated metabolic regulation rather than a uniform metabolic outcome. This Yin-Yang balance extends beyond tumor intrinsic metabolism to the tumor immune microenvironment, where SIRT1 and SIRT2 differentially shape immune cell metabolic fitness and antitumor function. By integrating these diverse and context-dependent roles, the Yin-Yang model provides a conceptual foundation for understanding how SIRT1 and SIRT2 coordinate metabolic adaptation in cancer and informs therapeutic strategies designed to restore metabolic balance. The following sections discuss how SIRT1 and SIRT2 regulate glucose, lipid, mitochondrial, and immune metabolism and examine the implications for cancer therapy.

## SIRT1 and SIRT2 in glucose metabolism: biosynthesis versus energy production

In glucose metabolism, SIRT1 and SIRT2 modulate key glycolytic enzymes and metabolic regulators, often converging on the same targets through distinct mechanisms 24. Below, we highlight five critical downstream effectors, including PKM2, PGAM1, HIF-1α, G6PD, and c-Myc, that are modulated by SIRT1 and SIRT2, illustrating their antagonistic balance as well as context-dependent cooperation roles in glucose metabolic reprogramming.

### 1. PKM2 regulation: SIRT1 driven biosynthesis versus SIRT2 driven energy production

Pyruvate kinase M2 (PKM2) is a glycolytic enzyme that catalyzes the conversion of phosphoenolpyruvate to pyruvate and functions as a rate-limiting step in glycolysis. Its activity depends on oligomeric state, with the tetramer being catalytically active and dimeric or monomeric forms favored in cancer cells, thereby promoting metabolic reprogramming and cell growth 25-27. SIRT1 and SIRT2 differentially modulate PKM2 through site-specific deacetylation, reflecting both Yin-Yang opposition and potential cooperation.

SIRT1 binds to and deacetylates PKM2 at Lys135 and Lys206, promoting dimerization 28. This shift toward a low activity dimer slows phosphoenolpyruvate to pyruvate conversion, creating a metabolic bottleneck that causes accumulation of upstream intermediates. These intermediates are shunted into anabolic pathways, elevating biosynthesis of cellular components such as nucleic acids and lipids 27. This metabolic reprogramming facilitates rapid tumor proliferation, consistent with the Warburg effect 27, 29. Thus, SIRT1 attenuates ATP generating glycolytic flux while promoting a biosynthetic glycolytic state characterized by reduced pyruvate kinase flux and enhanced shunting of intermediates into anabolic pathways.

Conversely, SIRT2 directly deacetylates PKM2 at Lys305, thereby activating its enzymatic activity and promoting tetramerization, favoring pyruvate production and increasing flux to TCA cycle and ATP generation. Loss of SIRT2 reverses these PKM2 dependent metabolic effects and, together with defects in DNA repair, cell cycle control, and lipid metabolism, supports tumor promoting phenotypes 30. Deacetylation of PKM2 by SIRT2 may therefore represent one key mechanism underlying the tumor suppressive functions of SIRT2 through regulation of glucose metabolism.

**Figure 1:**
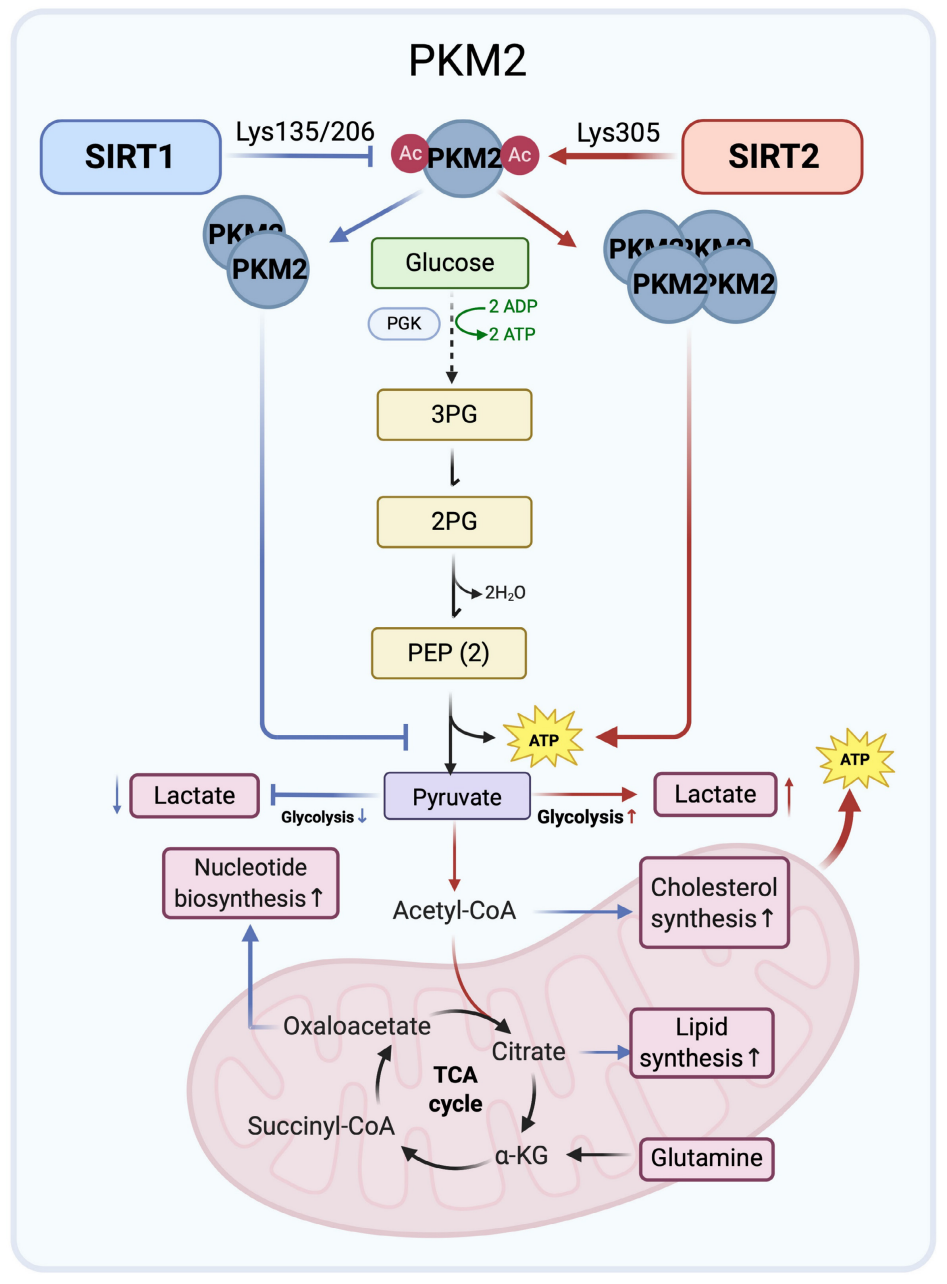
** Opposing regulation of PKM2 by SIRT1 and SIRT2 in glucose metabolism.** SIRT1 (blue arrows) deacetylates PKM2 at Lys135/Lys206 to promote dimerization and channel glycolytic intermediates into anabolic pathways. By contrast, SIRT2 (red arrows) targets Lys305 to induce tetramerization, enhancing ATP production and limiting glycolytic overflow. These opposing effects illustrate a Yin-Yang balance in cancer glucose metabolism. Created in BioRender. Fei, Y. (2026) https://BioRender.com/cmp7xbr.

In summary, SIRT1 and SIRT2 exert distinct controls over PKM2 that shape glycolytic flux in cancer cells. SIRT1 mediated deacetylation promotes dimerization, diverting glycolytic intermediates into anabolic pathways that support biosynthesis and tumor growth. In contrast, SIRT2 favors PKM2 tetramerization and enzymatic activation, enhancing ATP generation while limiting anabolic metabolism. These opposing yet complementary actions illustrate a Yin-Yang balance in which SIRT1 drives anabolic reprogramming and SIRT2 enhances energy efficiency. The acetylation state of PKM2 emerges as a critical molecular switch through which SIRT1 and SIRT2 orchestrate both antagonistic and cooperative control of glucose metabolism in cancer (Figure 1).

### 2. PGAM regulation: SIRT1 mediated glycolytic inhibition versus SIRT2 mediated glycolysis promotion

Phosphoglycerate mutases (PGAMs) are essential glycolytic enzymes that catalyze the interconversion of 3-phosphoglycerate (3-PG) and 2-phosphoglycerate (2-PG) in glycolysis 31. Two major members of the PGAM family are present in mammals, PGAM1 and PGAM2. PGAM1 is ubiquitously expressed and has been widely studied for its role in cancer metabolism, where it contributes to rerouting glucose flux toward biosynthetic pathways. By contrast, PGAM2, classically characterized as muscle specific, has only recently garnered attention for emerging functions in cancer biology. Evidence suggests that PGAM2 is subject to distinct regulatory mechanisms and influences tumor progression, potentially through interactions with oncogenic signaling pathways and modulation of glycolytic dynamics. Despite structural similarity to PGAM1, PGAM2 modulates cellular metabolism in distinct ways, and its impact on cancer cell metabolic phenotypes merits further investigation.

More recent research has revealed that SIRT1 functions as a stress responsive negative regulator of PGAM1. Under glucose restriction, SIRT1 levels rise, leading to reduced PGAM1 acetylation and suppression of its enzymatic activity. Mass spectrometry mapped acetylation sites to the C-terminal “cap” of PGAM1, a region previously implicated in catalytic control. Although the specific SIRT1 targeted lysines were not biochemically validated, functional assays with acetylation mimic mutants showed that acetylation enhances PGAM1 activity and glycolytic flux, whereas SIRT1 mediated deacetylation reduces its function. Diminished PGAM1 activity drives 3-PG accumulation, which in turn inhibits other metabolic pathways, including amino acid synthesis and the pentose phosphate pathway 32-34. These findings support a model in which SIRT1 represses glycolysis during energy stress by deacetylating and inactivating PGAM1 35.

Mass spectrometry-based proteomics have also implicated SIRT2 NAD⁺-dependent deacetylase in glycolytic control by identifying PGAM1 as a candidate substrate 36. Follow up studies confirmed SIRT2-PGAM1 interaction and showed that SIRT2 reduces PGAM1 activity, concomitantly restraining cell proliferation. Potential acetylation sites at Lys100, Lys106, Lys113, and Lys138 were mapped within the central region of PGAM1, though the specific lysine residue(s) directly targeted by SIRT2 remain undefined. Functional analyses using acetylation mimic mutants indicated that acetylation at these sites enhances PGAM1 enzymatic activity and glycolytic flux 37, supporting a model in which SIRT2 suppresses glycolysis through multi-site deacetylation of PGAM1.

Extending this finding, similar regulatory control was revealed for the PGAM1 related isozyme PGAM2. Proteomic evidence first implicated Lys100 as a conserved acetylation site shared by PGAM1 and PGAM2. Although the index peptide could derive from either isoform, subsequent mechanistic studies focused on PGAM2. SIRT2 directly binds PGAM2 and deacetylates Lys100, a residue critical for catalysis. Structural modeling and site-specific mutagenesis indicated that acetylation at Lys100 impairs PGAM2 activity, whereas SIRT2 mediated deacetylation relieves steric hindrance and facilitates formation of the catalytic p-His11 intermediate. Consistent with this, an acetylation mimic substitution at Lys100 abolishes enzymatic function 38, indicating that SIRT2 dependent deacetylation at this residue is required to maintain PGAM2 activity. Elevated PGAM2 activity accelerates 3-PG to 2-PG conversion and enhances glycolysis in mouse myoblasts 39. Thus, by deacetylating PGAM2 at Lys100, SIRT2 restores enzymatic activity, supports redox cofactor production, and promotes tumor cell proliferation, highlighting isoform specific control of PGAMs by SIRT2 rather than a uniformly inhibitory role in cancer metabolism.

**Figure 2:**
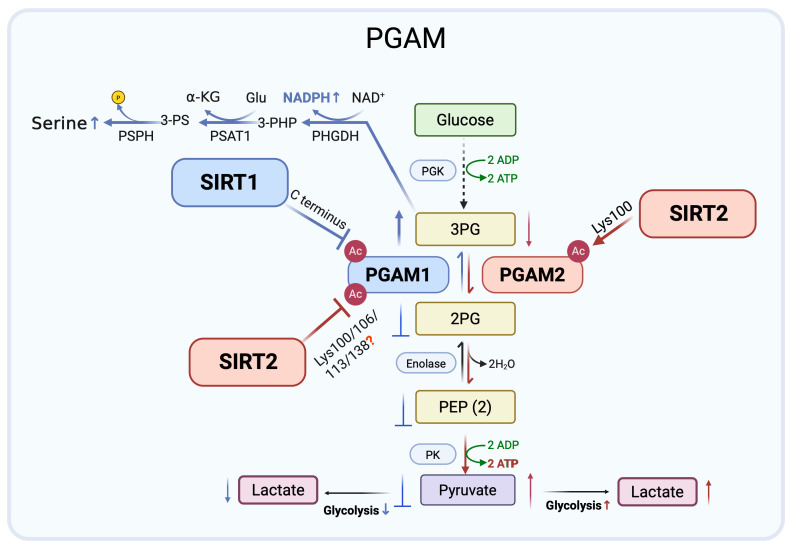
** Isoform specific regulation of PGAM1 and PGAM2 by SIRT1 and SIRT2.** SIRT1 (blue arrows) deacetylates PGAM1 in the C-terminal region under glucose limiting conditions, reducing enzymatic activity and glycolytic flux, thereby exerting a tumor suppressive effect. By contrast, SIRT2 (red arrows) inhibits PGAM1 through multi-site deacetylation (Lys100/106/113/138) but activates PGAM2 via deacetylation at Lys100, enhancing glycolysis, redox balance, and tumor cell proliferation. These opposing yet cooperative actions exemplify a Yin-Yang regulation of glycolysis by SIRT1 and SIRT2. Created in BioRender. Fei, Y. (2026) https://BioRender.com/cmp7xbr.

PGAM1 and PGAM2 are regulated by lysine acetylation, with SIRT1 and SIRT2 acting as key deacetylases that exert isoform and context-dependent effects. Under glucose limiting conditions, SIRT1 lowers PGAM1 activity, reducing glycolytic flux and conferring tumor suppressive effects. By contrast, SIRT2 inhibits PGAM1 but enhances PGAM2 activity through deacetylation at Lys100, restoring redox balance and supporting tumor cell proliferation. These opposing yet complementary actions resemble a Yin-Yang relationship, where SIRT1 restricts glycolysis while SIRT2 promotes metabolic adaptability. Collectively, these findings highlight the isoform specific and context-dependent control of PGAMs by SIRT1 and SIRT2, positioning them as both antagonistic and cooperative metabolic checkpoints and potential therapeutic targets in cancer (Figure 2).

### 3. G6PD regulation: SIRT2 dominant control of redox homeostasis

Glucose-6-phosphate dehydrogenase (G6PD), the rate limiting enzyme of the oxidative branch of the pentose phosphate pathway (PPP), sustains redox homeostasis and biosynthetic precursor production by generating NADPH 40. Its activity is tightly controlled by lysine acetylation deacetylation, with lysine 403 (Lys403) functioning as a critical regulatory site.

Accumulating evidence identifies SIRT2 as the principal regulator of G6PD through both enzymatic and post-translational mechanisms. SIRT2 directly interacts with G6PD and catalyzes deacetylation at Lys403, thereby enhancing enzymatic activity. This modification promotes the conversion of glucose-6-phosphate (G6P) to 6-phosphogluconate (6PG), increases PPP derived NADPH, and shifts glucose flux away from glycolysis 41-44. Consistently, the acetylation mimic mutant K403Q exhibits reduced G6PD activity and impaired antioxidant capacity, highlighting the functional importance of Lys403 deacetylation. Beyond direct enzymatic activation, SIRT2 also stabilizes G6PD protein under stress conditions. By limiting ubiquitination and promoting SUMO1 modification, SIRT2 prolongs G6PD protein half-life and sustains NADPH production during oxidative or metabolic stress 45. These combined mechanisms position SIRT2 as a central controller of PPP flux and redox balance.

**Figure 3:**
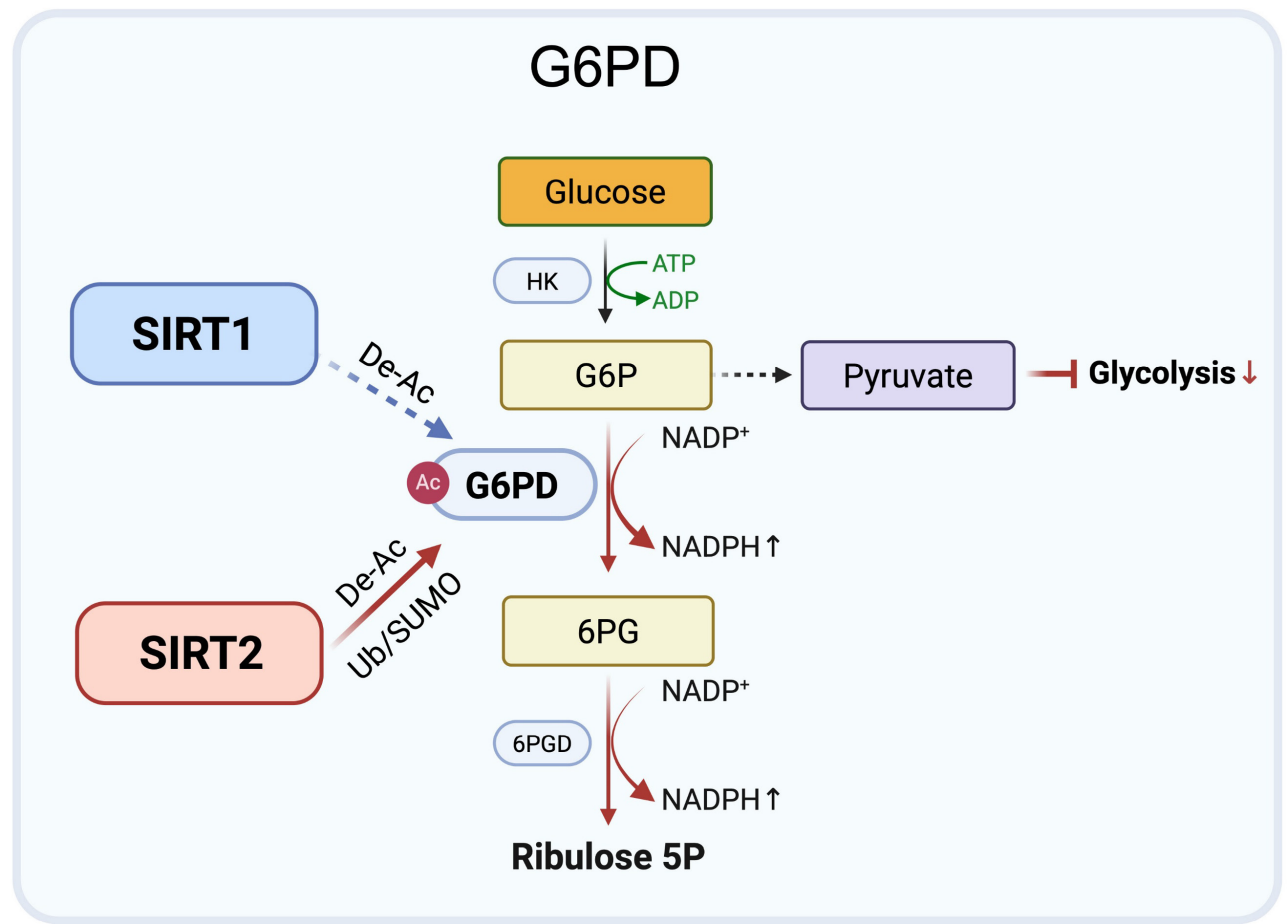
** Complementary control of G6PD by SIRT1 and SIRT2 under metabolic stress.** SIRT2 (red arrows) primarily regulates G6PD by deacetylating Lys403 (De-Ac) and stabilizing the protein via reduced ubiquitination (Ub) and enhanced SUMO1 modification, sustaining NADPH production and redox balance. SIRT1 (blue arrows) also deacetylates Lys403 under nutrient deprivation or oxidative stress, serving as a supportive regulator. Together, they maintain redox homeostasis and metabolic adaptation in cancer cells. Created in BioRender. Fei, Y. (2026) https://BioRender.com/cmp7xbr.

Overall, current evidence supports a SIRT2 dominant model of G6PD regulation, in which SIRT2 promotes both deacetylation and stabilization of G6PD to maintain redox homeostasis in tumor cells exposed to metabolic stress. In contrast, the contribution of SIRT1 to direct G6PD regulation remains less well defined and appears to be context-dependent. Rather than acting as a primary regulator, SIRT1 may provide supportive regulation under conditions such as nutrient limitation or oxidative pressure. This model suggests distinct regulatory roles, with SIRT2 providing primary control of G6PD activity, while other Sirtuins may fine tune redox adaptation in specific stress contexts (Figure 3).

### 4. HIF-1α regulation: context-dependent roles of SIRT1 and SIRT2 in stability and activity

Hypoxia inducible factor 1-alpha (*HIF-1α*) is a key transcription factor that enables cellular adaptation to low oxygen 46. During hypoxia, *HIF-1α* is stabilized, translocates to the nucleus, dimerizes with *HIF-1β*, and activates target genes including *GLUT1*, *HK2*, *LDHA*, and *PDK1*, promoting glycolysis, angiogenesis, erythropoiesis, and cell survival 47-49. HIF-1α function is governed by post-translational modifications, including acetylation and hydroxylation, that regulate its protein stability and transcriptional activity. Induction of glycolytic enzymes by HIF-1α is central to metabolic reprogramming, particularly in cancers exhibiting the Warburg effect. Identifying upstream modulators of HIF-1α stability and transactivation is therefore essential for understanding glucose metabolic adaptation under hypoxia.

SIRT1 and SIRT2 exert distinct, context-dependent influences on HIF-1α stability and activity, with subsequent effects on downstream glycolytic pathways. Recent work shows that SIRT1 regulates the phenotypic and metabolic reprogramming of myeloid derived suppressor cells (MDSCs). In the context of non-tumor biology, immune cells represent a defined subset in which nutrient availability, oxygen tension, and genetic perturbations can be precisely controlled, enabling mechanistic analysis of SIRT1-HIF-1α regulation. A key finding is that myeloid specific deletion of SIRT1 increases glycolytic activity, an effect abolished either by rapamycin mediated inhibition of mTOR or by genetic deletion of HIF-1α 50. These findings indicate that SIRT1 restrains glycolysis via an mTOR-HIF-1α pathway, with HIF-1α acting downstream of mTOR signaling 51, 52. The authors termed this the “SIRT1-mTOR/HIF-1α glycolytic pathway,” reflecting a defined regulatory hierarchy. Consistent with this model, rapamycin only partially reduces the SIRT1 knockout induced upregulation of HIF-1α 50, whereas mTOR deficiency normalizes the elevated HIF-1α levels in SIRT1 deficient CD4⁺ T cells and restores glycolytic activity toward baseline 53. These genetic observations support a SIRT1-mTOR-HIF-1α regulatory axis, in which SIRT1 negatively regulates glycolysis by inhibiting mTOR activity and thereby limiting HIF-1α function. Although no direct SIRT1-HIF-1α interaction was detected, the loss of glycolytic and transcriptional changes upon simultaneous deletion of SIRT1 and mTOR places SIRT1 further upstream of the mTOR-HIF-1α pathway.

By contrast, a study using CD11c specific SIRT1 knockout mice found that SIRT1 deficiency in dendritic cells (DCs) increased HIF-1α levels that were not corrected by rapamycin mediated mTOR inhibition 54. This suggests that SIRT1 can also regulate HIF-1α through an mTOR independent route, though the mechanism remains unclear. These divergent results indicate that SIRT1-HIF-1α regulation varies across immune cell types, being evident in Th9 cells and MDSCs but not in DCs. The extent of mTOR involvement may therefore reflect cell type specific metabolic programming and context-dependent control of HIF-1α stability.

SIRT1 also modulates HIF-1α protein stability in a context-dependent manner beyond immune cells. To distinguish immune intrinsic regulation from effects observed in non-immune systems, several studies have examined non-tumor epithelial cells under controlled normoxic or hypoxic conditions. In HK2 renal epithelial cells, SIRT1 loss leads to an increase in HIF-1α protein levels 55. In contrast, studies in several tumor cell lines, including HeLa (cervical cancer), Hep3B, HepG2, and SK-Hep-1 (hepatocellular carcinoma), HT1080 (fibrosarcoma), and SiHa (cervical cancer), consistently show that genetic or pharmacological inhibition of SIRT1 reduces HIF-1α protein levels 56-58. In cancer cells, SIRT1 appears to stabilize HIF-1α, most likely through deacetylation dependent mechanisms that enhance both its stability and transcriptional activity, with Lys709 identified as the relevant acetylation site 56, 58, 59. These opposing outcomes underscore the strong dependence of SIRT1 mediated HIF-1α regulation on cellular context and microenvironmental conditions. The contrasting effects observed in tumor versus normal cells raises an important, unexplored question, and highlights the need to determine how SIRT1 influences hypoxia signaling under physiological and pathological conditions.

In addition to its effect on protein stability, SIRT1 also directly interacts with and deacetylates HIF-1α to regulate its activity in tumor cell under hypoxic conditions. The acetylation site Lys674 is critical for HIF-1α function; SIRT1 mediated deacetylation at Lys674 suppresses transcription of glycolytic genes such as *PDK1* by limiting recruitment of the coactivator p300 60. This mechanism promotes a shift from glycolysis toward oxidative phosphorylation, particularly under normoxic or NAD⁺ replete conditions.

By comparison, the impact of SIRT2 on HIF-1α under hypoxia remains controversial and appears to depend on how SIRT2 is inhibited. SIRT2 has been reported to suppress HIF-1α accumulation by deacetylating Lys709, thereby promoting hydroxylation and PHD2 dependent degradation 61. Stabilization of HIF-1α under hypoxia upon SIRT2 loss was further supported by siRNA mediated silencing in HeLa cells and by SIRT2 knockout in chicken DT40 and human Nalm-6 cells 62. In contrast, pharmacological inhibition of SIRT2 with AK-1 unexpectedly enhanced HIF-1α degradation in a VHL dependent manner 63. The authors proposed that genetic loss of SIRT2 may preserve a multi protein complex required for HIF-1α homeostasis, whereas chemical inhibition disrupts complex formation while impairing enzymatic activity. These discrepancies indicate that SIRT2 regulation of HIF-1α depends on the mode of inhibition and experimental context, requiring further investigation.

Taken together, current evidence indicates that SIRT1 and SIRT2 exert opposing yet complementary control over HIF-1α signaling. SIRT1 can either suppress or stabilize HIF-1α in a context-dependent manner, acting through pathways such as mTOR signaling or by direct deacetylation at Lys674 and Lys709. In immune cells, SIRT1 often inhibits HIF-1α activity and glycolysis, whereas in tumor cells it tends to stabilize HIF-1α and promote glycolytic reprogramming. By contrast, SIRT2 generally destabilizes HIF-1α via deacetylation at Lys709 and PHD2 mediated degradation, though conflicting results with pharmacological inhibitors point to modality and context-dependent effects. These features support a Yin-Yang model in which SIRT1 and SIRT2 can oppose one another to tune HIF-1α signaling, yet may also converge in specific settings to coordinate redox balance and metabolic adaptation.

### 5. c-Myc regulation: SIRT1 mediated destabilization versus SIRT2 mediated stabilization

*c-Myc* is an oncogenic transcription factor frequently activated in cancer. It enhances glucose uptake and lactate production by driving the expression of glycolysis related genes, including *LDHA, ENO1, PKM2, and GLUT1* 64, 65. This metabolic reprogramming, consistent with the Warburg effect, promotes aerobic glycolysis under normoxia to supply biosynthetic precursors and energy. Given the critical role of c-Myc in driving glycolysis in cancer cells, understanding how its stability and activity are controlled has become a major focus. Emerging evidence shows that SIRT1 and SIRT2 modulate c-Myc abundance and function, linking c-Myc regulation to cancer metabolism and epigenetic control.

Studies indicate that the interaction between SIRT1 and c-Myc may be context-dependent. Yuan et al. reported that c-Myc transcriptionally upregulates SIRT1, and SIRT1 mediated deacetylation reduces c-Myc protein stability and inhibits transcription of glycolytic genes such as *LDHA*, thereby preventing cellular transformation and tumorigenesis 66. By contrast, Menssen et al. found that SIRT1 overexpression prolonged c-Myc half-life, whereas SIRT1 loss decreased c-Myc stability by reducing K63 linked polyubiquitination 67. Although both studies agree that SIRT1 deacetylates c-Myc, they reached differing conclusions regarding whether deacetylation affects c-Myc protein stability. In the Menssen et al. study, the K323R mutant did not affect the activity of c-Myc, implying that Lys323 may not mediate transcriptional regulation in their experimental system. One key difference between the two studies lies in how c-Myc half-life was measured: Yuan et al. used cycloheximide chase assays in HEK293T cells, whereas Menssen et al performed [35S]-methionine pulse labeling in MEFs. Differences in assay methods and cell types likely explain the inconsistent results for c-Myc half-life and stability 68-70. Yuan et al. concluded that Lys323 is a critical site through which SIRT1 regulates c-Myc stability, while Menssen et al. did not exclude the possibility that additional lysine residues serve as SIRT1 deacetylation targets. At present, no consensus has emerged on whether SIRT1 mediated deacetylation stabilizes or destabilizes c-Myc; the outcome likely depends on cellular context, c-Myc expression level, and the presence of other SIRT1 targets or cofactors.

SIRT2 has been more consistently implicated in pro-tumorigenic functions, whereas SIRT1 is generally not considered tumor promoting. Mechanistically, SIRT2 deacetylates histone H4 at lysine 16 (H4Lys16Ac) within the *NEDD4* core promoter, repressing *NEDD4* transcription and lowering NEDD4 protein abundance. Reduced NEDD4 attenuates c-Myc ubiquitination and proteolysis, thereby stabilizing c-Myc. Pharmacological inhibition of SIRT2 with small molecules such as AC-93253 or Salermide restores NEDD4 expression, promotes c-Myc degradation, and suppresses tumor cell proliferation in pancreatic cancer and neuroblastoma models 71. SIRT2 inhibition also decreases Aurora A kinase, which colocalizes with the c-Myc N-terminal transactivation domain to shield c-Myc from ubiquitin mediated degradation and enhance its transcriptional activity 71-74. Consistent with these results, the SIRT2 selective inhibitor TM induces c-Myc ubiquitination and degradation and suppresses cancer cell growth 75. These findings support SIRT2 inhibition as a potential therapeutic strategy in c-Myc driven malignancies. In cholangiocarcinoma, SIRT2 inhibition reduces c-Myc and phosphorylated PDHA1 (p-PDHA1), and c-Myc knockdown similarly lowers p-PDHA1, suggesting that the SIRT2-c-Myc axis promotes metabolic reprogramming by enhancing PDHA1 phosphorylation, shifting metabolism from the TCA cycle toward glycolysis and contributing to the Warburg effect 76.

SIRT1 and SIRT2 exert divergent, and at times opposing, effects on c-Myc stability and function. SIRT1 deacetylates c-Myc at Lys323 in a context-dependent manner, reducing its stability and transcriptional activity. By contrast, SIRT2 indirectly stabilizes c-Myc by repressing NEDD4 or maintaining Aurora A kinase, thereby limiting c-Myc ubiquitination and proteolysis and promoting glycolytic metabolism. These opposing actions reflect a Yin-Yang dynamic, in which SIRT1 counterbalances c-Myc activity, whereas SIRT2 reinforces its oncogenic function, with both contributing to metabolic plasticity. Through post-translational regulation of key metabolic enzymes and transcription factors, SIRT1 and SIRT2 modulate glycolysis and glucose flux through post-translational regulation of key metabolic enzymes and transcription factors within a multilayered network. By differentially targeting PKM2, PGAM1, G6PD, HIF-1α, and c-Myc, these Sirtuins reprogram glucose metabolism to support tumor adaptation and survival. This complementary Yin-Yang interplay highlights the central roles of SIRT1 and SIRT2 in cancer metabolic reprogramming and highlights potential therapeutic entry points (Table 1).

## SIRT1 and SIRT2 in lipid metabolism: storage versus catabolism

Dysregulated lipid metabolism is a hallmark of cancer, supporting rapid proliferation and adaptation to metabolic stress. Emerging evidence indicates that SIRT1 and SIRT2 coordinate lipid metabolic programs by converging on ATP citrate lyase (ACLY), which generates cytosolic acetyl-CoA from citrate and serves as a central metabolic hub.

In tumor cells, SIRT2 deacetylates ACLY at Lys540, Lys546, and Lys554, promoting its ubiquitination and proteasomal degradation. This reduces acetyl-CoA availability and suppresses de novo lipogenesis, thereby restraining lipid driven tumor growth. Under high glucose conditions, ACLY acetylation is enhanced, leading to protein stabilization, increased lipid synthesis, and tumor progression. Loss of SIRT2 results in ACLY accumulation and elevated lipogenesis, highlighting SIRT2 as a metabolic brake in cancer cells 77. By contrast, in non-tumor settings such as renal ischemia reperfusion injury, SIRT1 promotes *ACLY* transcription via deacetylation of SP1, enhancing fatty acid oxidation and conferring protection against fibrosis 78. Taken together, SIRT1 and SIRT2 regulate ACLY in a complementary yet context-dependent manner. SIRT1 increases ACLY expression in non-cancerous tissues under metabolic stress, supporting adaptive energy metabolism. In contrast, SIRT2 promotes ACLY protein degradation in cancer cells, thereby limiting lipogenesis and restraining tumor-associated metabolic activity. Through these distinct roles, Sirtuins drive metabolic reprogramming and represent potential therapeutic targets in both cancer and metabolic diseases.

Downstream of ACLY, acetyl-CoA is partitioned into three major metabolic fates. First, acetyl-CoA fuels lipogenesis, providing substrates for fatty acid and cholesterol synthesis. SIRT1 and SIRT2 regulate the *FOXO* family, particularly *FOXO1 and FOXO3*, which suppress tumor promoting lipid accumulation by repressing lipogenic transcription factors such as *PPARγ and SREBP1*. In hepatic and adipose tissues, nuclear SIRT1 promotes lipid catabolism primarily through transcriptional reprogramming. By deacetylating FOXO1 and FOXO3 in the nucleus, SIRT1 enhances the expression of lipolytic enzymes such as adipose triglyceride lipase (*ATGL*) while repressing key lipogenic regulators, including *PPARγ* and *SREBP1*. This coordinated transcriptional control limits lipid accumulation under metabolic stress 79. Notably, this anti-lipogenic role of SIRT1 is context-dependent. In endometrial cancer cells, SIRT1 has been reported to promote lipid biosynthesis by upregulating *SREBP1* and its downstream target *FASN*, thereby facilitating tumor growth through enhanced lipogenesis 80.

However, SIRT2 exerts its anti-lipogenic function mainly in the cytoplasm. SIRT2 deacetylates FOXO1, strengthening its repressive interaction with PPARγ and favoring FOXO1 nuclear retention. This mechanism maintains transcriptional repression of adipogenic programs, preventing adipocyte differentiation and lipid storage 81, 82.

Second, acetyl-CoA supports histone and protein acetylation, linking metabolic state to epigenetic and transcriptional regulation. By modulating the balance between acetyltransferase activity and NAD⁺ dependent deacetylation, Sirtuins couple nutrient availability to chromatin accessibility and gene expression programs.

Third, acetyl-CoA availability influences mitochondrial oxidative metabolism. SIRT1 directly deacetylates PGC-1α, converting it from an inactive to an active state and thereby promoting fatty acid oxidation (FAO) and oxidative phosphorylation (OXPHOS). This direct SIRT1-PGC-1α axis enhances mitochondrial energy output and supports metabolic adaptation under nutrient stress 83-85.

In addition to this direct regulation, SIRT1 can further reinforce mitochondrial oxidation through the AMPK pathway. By deacetylating LKB1, SIRT1 enhances AMPK phosphorylation, leading to suppression of lipogenic enzymes such as fatty acid synthase (FASN) and stimulation of FAO 86. AMPK, in turn, can phosphorylate and activate SIRT1 by releasing it from the endogenous inhibitor DBC1, forming a positive feedback loop that amplifies SIRT1 dependent metabolic reprogramming 87. Consistent with this model, pharmacological activation of SIRT1 by SCIC2.1 in hepatocellular carcinoma under glucose deprivation promotes FAO and mitochondrial function via AMPK-PGC-1α signaling while inhibiting lipogenesis 88.

Several studies suggest that SIRT2 may also modulate the AMPK axis in a context-dependent manner, although its role in AMPK driven lipid metabolism remains less defined. SIRT2 mediated activation of LKB1 has been reported in cardiomyocytes 89, whereas inhibition of AMPK signaling has been observed in liver failure models 90. Overall, while SIRT1 consistently promotes lipid catabolism and mitochondrial resilience to metabolic stress, the contribution of SIRT2 to AMPK signaling appears variable and tissue specific.

**Figure 4:**
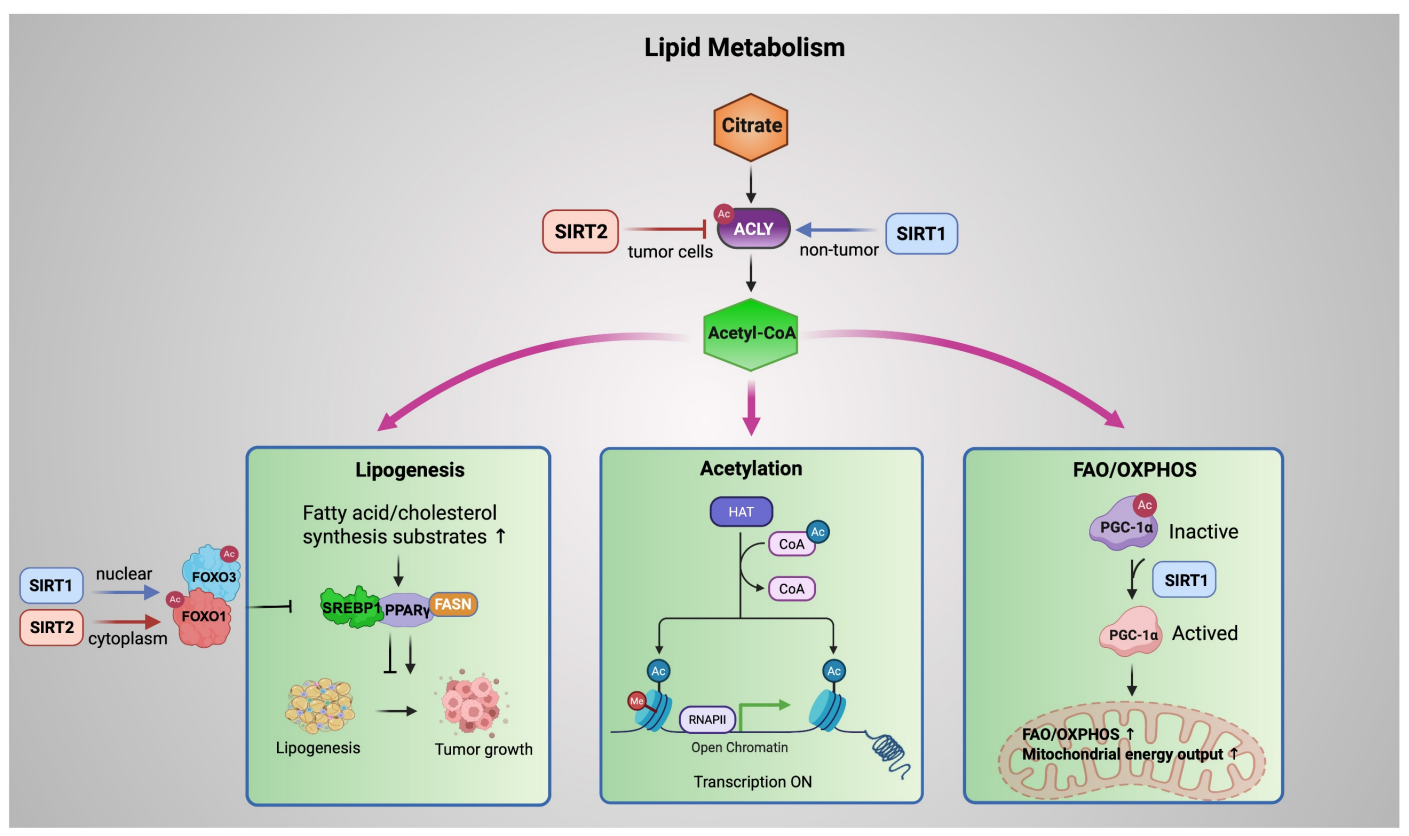
** SIRT1 and SIRT2 coordinate lipid metabolism via an ACLY-acetyl-CoA metabolic fork.** ACLY generates acetyl-CoA that fuels lipogenesis, acetylation dependent regulation, and mitochondrial FAO/OXPHOS. SIRT2 (red) restricts tumor-associated lipogenesis by promoting ACLY deacetylation and degradation, whereas SIRT1 (blue) supports metabolic adaptation in non-tumor contexts. SIRT1 (nuclear) and SIRT2 (cytoplasmic) deacetylate FOXO factors to suppress SREBP1/PPARγ driven lipogenic programs, while SIRT1 directly activates PGC-1α to enhance mitochondrial oxidative metabolism. Blue arrows denote SIRT1 mediated regulation; red arrows denote SIRT2 mediated regulation. Created in BioRender. Fei, Y. (2026) https://BioRender.com/cmp7xbr.

Collectively, these pathways form an ACLY-acetyl-CoA centered metabolic fork, in which SIRT1 and SIRT2 exert spatially and contextually distinct control over lipid synthesis, acetylation dependent regulation, and mitochondrial oxidation. Through this integrated network, Sirtuins balance lipid storage and catabolism, shaping metabolic plasticity in cancer and non-tumor tissues (Figure 4).

## SIRT1 and SIRT2 in mitochondrial metabolism: energy output versus integrity

Mitochondrial metabolism is a central hub that coordinates cellular energy production, biosynthesis, and epigenetic regulation 91. Growing evidence indicates that SIRT1 and SIRT2 are key regulators of this network through distinct yet complementary mechanisms, maintaining mitochondrial energy homeostasis through coordinated control.

Peroxisome proliferator activated receptor gamma coactivator 1-alpha (*PGC-1α*), a master transcriptional coactivator of *PPARγ*, plays a central role in regulating mitochondrial biogenesis and oxidative metabolism, particularly in hepatic tissue. Its expression and activity are modulated by nutrient and hormonal signals (e.g., glucagon, glucocorticoids) and by post-translational modifications 92. During fasting or exercise, SIRT1 directly deacetylates PGC-1α, enhancing its transcriptional activity and promoting expression of genes involved in mitochondrial fatty acid oxidation and gluconeogenesis, thereby supporting energy homeostasis 93. Similarly, PGC-1α is deacetylated and activated by SIRT1 and acetylated and inhibited by GCN5 by resveratrol. AMPK modulates SIRT1 activity by regulating the intracellular NAD^+^ level, thereby increasing the expression of genes governing oxidative phosphorylation, fatty acid oxidation, and mitochondrial biogenesis 94. Moreover, SIRT1 localizes not only to the nucleus but also to mitochondria, where it interacts with TFAM and PGC-1α at the mitochondrial nucleoid and may directly regulate mtDNA transcription and mitochondrial gene expression, highlighting their cooperative role in sustaining mitochondrial function 11.

Beyond normal physiology, dysregulation of the SIRT1-PGC-1α axis contributes to cancer metabolism. In hepatocellular carcinoma (HCC), SIRT1 enhances mitochondrial energy metabolism via PGC-1α activation, thereby promoting cancer cell invasion and metastasis in vitro and in vivo 95. In diffuse large B cell lymphoma (DLBCL), SIRT1 mediated deacetylation of PGC-1α supports Adriamycin resistance by promoting mitochondrial biogenesis, increasing expression of mitochondrial DNA encoded genes such as *COX1, ND1, and ND6*, and boosting ATP production 96. This axis has also been linked to hypoxia induced chemoresistance in non-small cell lung cancer (NSCLC), where the SIRT1-PGC-1α-PPARγ signaling pathway helps maintain cellular energy under stress 97. Furthermore, pharmacological activation of the SIRT1-PGC-1α pathway shows therapeutic potential: bouchardatine (Bou) elevates NAD⁺/NADH ratios to activate the SIRT1-PGC-1α-UCP2 axis, shifting metabolic preference toward oxidative phosphorylation and suppressing colorectal cancer growth 98. Similarly, diallyl trisulfide (DATS) reverses cisplatin resistance in ovarian cancer by upregulating the AMPK-SIRT1-PGC-1α axis, thereby increasing reactive oxygen species (ROS) production and apoptosis 99.

SIRT2 also deacetylates PGC-1α and localizes to the inner mitochondrial membrane, where it increases mitochondrial respiration by deacetylating mitochondrial proteins. Moreover, SIRT2 modulates PGC-1α to reduce intracellular ROS levels and increase resistance to oxidative stress, thereby preserving mitochondrial integrity and supporting cell survival under stress conditions 100, 101.

**Figure 5:**
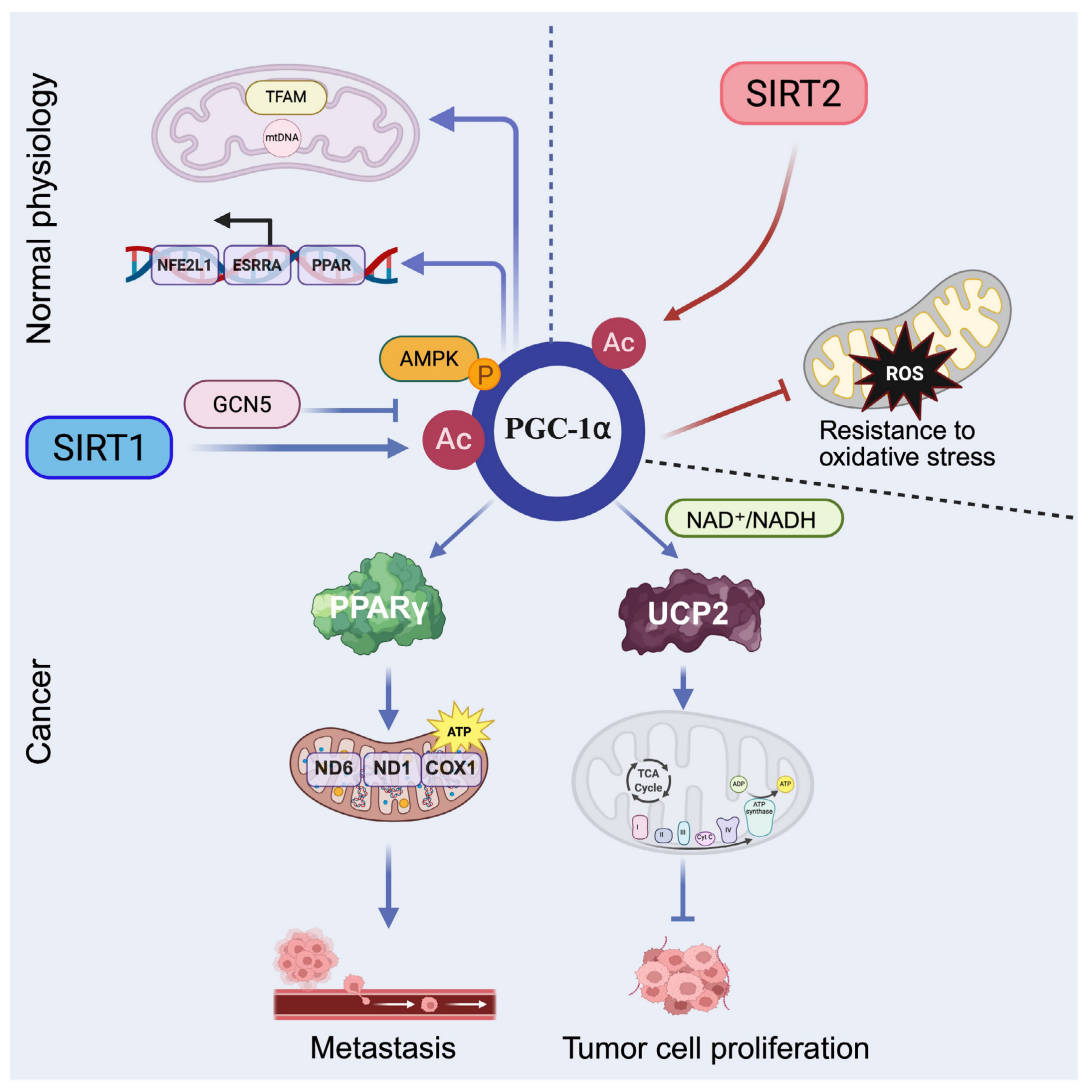
** Regulation of mitochondrial metabolism by SIRT1 and SIRT2.** SIRT1 (blue arrows) promotes PGC-1α mediated mitochondrial fatty acid oxidation, gluconeogenesis, biogenesis, and oxidative phosphorylation, thereby enhancing ATP production. SIRT2 (red arrows) regulates PGC-1α to reduce intracellular ROS and increase resistance to oxidative stress. Dashed arrows represent regulation under physiological conditions, whereas solid arrows indicate effects observed in tumor settings. Created in BioRender. Fei, Y. (2026) https://BioRender.com/d6fcsxa.

Overall, the SIRT1/2-PGC-1α axis integrates energy production with stress adaptation in mitochondrial metabolism. SIRT1 enhances mitochondrial biogenesis and oxidative phosphorylation by deacetylating PGC-1α, thereby boosting energy output and, under metabolic stress, supporting tumor progression. By contrast, SIRT2 helps preserve mitochondrial function by deacetylating mitochondrial proteins, reducing oxidative stress, and maintaining cell survival in adverse conditions. These opposing yet complementary actions are consistent with a Yin-Yang mode of regulation: SIRT1 drives energy production, while SIRT2 safeguards mitochondrial integrity. Together, they tune mitochondrial metabolism to influence tumor adaptation, therapy resistance, and metabolic vulnerability (Figure 5).

Given the breadth of metabolic pathways and experimental systems discussed above, it is important to assess the strength of these conclusions across different model types and perturbation strategies. Considering the diversity and complexity of the available evidence, key SIRT1 and SIRT2 regulated metabolic functions and their experimental support are summarized in Table 2 to aid reader evaluation.

## SIRT1 and SIRT2 in the tumor immune microenvironment: context-dependent immune modulation and metabolic checkpoint control

The tumor immune microenvironment (TME) is a complex network of tumor, immune, and stromal cells that collectively shape tumor progression and therapeutic response. Metabolic competition and immunosuppressive signaling within the TME often impair effector immune cell function, contributing to immune evasion and therapy resistance 102, 103.

SIRT1 and SIRT2 are key metabolic regulators with distinct, and at times opposing, effects on immune function in the TME. SIRT1 exhibits context-dependent dual roles in immune metabolism and tumor immune evasion. In colorectal cancer, tumor-intrinsic SIRT1 drives glucolipid metabolic reprogramming and enhances immunosuppressive Treg activity by increasing CX3CL1 secretion, which activates the CX3CR1-SATB1/BTG2 axis and promotes differentiation of highly suppressive TNFRSF9⁺ Tregs 104. By contrast, spatial proteomic and functional studies in melanoma associate SIRT1 expression with increased CD8⁺ T cell infiltration and elevated IFN-γ/CXCL9/CXCL10, supporting the efficacy of immune checkpoint blockade 105. Mechanistically, SIRT1 modulates immune metabolism through NAD⁺ dependent deacetylation of key transcription factors such as *NF-κB and HIF-1α* in immune cells, as well as by regulating the secretion of cytokines like IL-12 and TGF-β, thereby influencing the differentiation and function of T cells and dendritic cells 106. Consistent with these context specific roles, preclinical studies have shown that pharmacologic inhibition of SIRT1 can reprogram tumor immune signaling and enhance sensitivity to PD-1 blockade in selected tumor settings. In these models, SIRT1 inhibition was associated with changes in PD-L1 expression and subcellular distribution, along with attenuation of immunosuppressive programs within the tumor microenvironment 107, 108. Moreover, combination strategies incorporating SIRT1 inhibitors, such as EX-527, in combination with epigenetic or transcriptional modulators including the BET inhibitor JQ-1, have been shown to further reduce tumor growth in vivo 109. These data support SIRT1 targeting as a conditional immunomodulatory strategy that cooperates with immune checkpoint inhibition and selected metabolic or transcriptional interventions, rather than as a universal immune activator 110.

**Figure 6:**
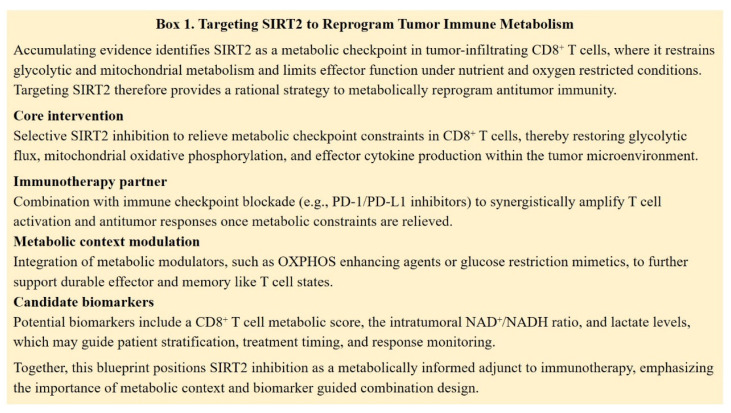
Targeting SIRT2 to reprogram tumor immune metabolism.

In contrast, SIRT2 functions predominantly as a suppressor of antitumor T cell immunity. SIRT2 is upregulated in tumor infiltrating lymphocytes and restrains T cell metabolic reprogramming by deacetylating enzymes involved in glycolysis, tricarboxylic acid (TCA) cycle, fatty acid oxidation (FAO), and glutaminolysis. Genetic or pharmacologic loss of SIRT2 enhances glycolysis and oxidative phosphorylation in CD8⁺ T cells, improving proliferation, cytokine production, and cytotoxicity. These observations identify SIRT2 as a metabolic checkpoint that limits T cell fitness in the TME and suggest that selective SIRT2 inhibition could restore effective antitumor responses in metabolically suppressed immune cells 111. Rather than acting as an independent immunotherapy, SIRT2 inhibition is more likely to function as a context-dependent metabolic modulator. By relieving metabolic limitations in CD8⁺ T cells, including constraints on glycolytic capacity and mitochondrial function, SIRT2 blockade has the potential to support T cell effector activity and responsiveness to immune checkpoint inhibition. Such effects are expected to be most relevant within metabolically restrictive tumor microenvironments. Beyond immune cells, multiple preclinical studies further support the role of SIRT2 inhibition as a cooperative partner for metabolic and signaling targeted therapies. The selective SIRT2 inhibitor SirReal2 has been shown to enhance the antitumor efficacy of PI3K/mTOR inhibition in acute myeloid leukemia, highlighting functional crosstalk between SIRT2 and growth factor driven metabolic pathways 112. In solid tumors, metabolic reprogramming strategies that enhance mitochondrial oxidation by dichloroacetic acid synergize with SIRT2 inhibitors, including sirtinol and AGK2, to suppress tumor growth in non-small cell lung cancer 113. Moreover, recent studies in preclinical colorectal cancer models have demonstrated that targeting SIRT2 induces MLH1 deficiency, increases tumor immunogenicity, and potentiates antitumor immune responses 114. Collectively, these findings support a rational combination framework in which SIRT2 inhibitors are integrated with immune checkpoint blockade and selected metabolic modulators to counteract tumor-associated metabolic constraints and improve therapeutic efficacy (Figure 6).

Taken together, SIRT1 and SIRT2 exemplify a Yin-Yang mode of immuno metabolic regulation in the tumor immune microenvironment. SIRT1 exerts context-dependent effects that can either promote immune suppression or support antitumor immunity, whereas SIRT2 functions more consistently as a metabolic checkpoint that constrains T cell fitness. Their complementary roles highlight Sirtuins dependent metabolic pathways as targets for restoring immune balance in cancer.

## Spatial flexibility and autophagy mediated metabolic adaptation by SIRT1 and SIRT2

Tumor cells reside in a metabolically challenging microenvironment characterized by hypoxia, nutrient limitation, and fluctuating energy supply. These conditions require rapid and reversible adaptive mechanisms that extend beyond transcriptional reprogramming. In this setting, SIRT1 and SIRT2 display spatially flexible functions that allow them to coordinate metabolic regulation across cellular compartments and converge on autophagy as a key adaptive process.

### 1. Spatially distinct and non-canonical functions of SIRT1 and SIRT2

SIRT1 is classically defined as a nuclear deacetylase that regulates transcriptional programs controlling mitochondrial function, oxidative metabolism, and stress responses. However, accumulating evidence indicates that SIRT1 also operates in the cytoplasm, particularly under metabolic stress 115. In this compartment, SIRT1 directly modulates metabolic enzymes and autophagy related proteins, enabling rapid metabolic adjustment independent of de novo gene expression 116, 117. This non-canonical activity positions SIRT1 as an immediate responder to energetic stress.

By contrast, SIRT2 is predominantly cytoplasmic and is best known for regulating glycolysis, cytoskeletal organization, and cell cycle progression. Notably, SIRT2 can translocate to the nucleus in a stress or cell cycle cues 118, 119. Nuclear SIRT2 participates in chromatin associated processes and genome maintenance, thereby linking metabolic status to cell cycle control and stress tolerance 120. Together, these findings indicate that the biological consequences of SIRT1 and SIRT2 is determined not only by substrate specificity but also by dynamic subcellular localization.

### 2. Autophagy as a metabolic integration node regulated by SIRT1 and SIRT2

Autophagy is a fundamental metabolic process that supports energy homeostasis by recycling intracellular components during stress. In cancer, autophagy functions as a flexible metabolic buffer that sustains survival under adverse conditions. SIRT1 and SIRT2 regulate autophagy through distinct yet interconnected mechanisms.

SIRT1 primarily promotes autophagy initiation through direct deacetylation of core autophagy machinery. SIRT1 has been shown to associate with and deacetylate ATG5, ATG7, and LC3/ATG8, which is required for efficient autophagy induction under nutrient deprivation 117. In addition, SIRT1 deacetylates LC3 at lysine residues Lys49 and Lys51, enabling its nucleocytoplasmic redistribution and subsequent participation in autophagosome formation 121. SIRT1 also regulates autophagosome maturation by deacetylating Beclin 1 (BECN1) at Lys430 and Lys437, counteracting inhibitory acetylation and facilitating autophagic progression 122. Through these coordinated deacetylation events, SIRT1 enhances autophagic flux and promotes nutrient recycling, thereby supporting metabolic resilience and tumor cell survival under energetic stress.

In contrast, SIRT2 functions as a context-dependent modulator of autophagic flux by acting at multiple stages of the autophagy pathway. Under nutrient deprivation, SIRT2 has been reported to promote autophagy initiation through deacetylation of ATG4B at lysine 39 (Lys39), which enhances ATG4B protease activity and facilitates LC3 processing 123. At later stages of the pathway, SIRT2 activity is tightly linked to autophagosome trafficking and cargo clearance through its established role in deacetylating α-tubulin, a key determinant of microtubule stability 13, 124. In macrophages, elevated SIRT2 expression directly reduces α-tubulin acetylation and impairs autophagy clearance, whereas pharmacologic inhibition of SIRT2 restores autophagic flux in this setting. By reducing microtubule acetylation, SIRT2 can constrain autophagosome transport and limit autophagic clearance in specific cellular contexts 125. Through this stage specific regulation, SIRT2 fine tunes autophagic flux to prevent excessive or prolonged catabolic activity, thereby maintaining metabolic balance under stress conditions.

Within the Yin-Yang framework, SIRT1 biases the system toward autophagy activation to restore metabolic balance, whereas SIRT2 fine tunes autophagic flux by regulating processing efficiency and intracellular trafficking. This dynamic interplay ensures that autophagy remains adaptive rather than deleterious, thereby enabling cancer cells to maintain metabolic homeostasis while preventing excessive energy depletion in a fluctuating tumor microenvironment.

Together, the spatial and functional plasticity of SIRT1 and SIRT2 underscores the importance of viewing cancer metabolism as an integrated and stress responsive network. This conceptual framework provides a basis for understanding how metabolic adaptation is coordinated at the cellular level and informs therapeutic strategies targeting the SIRT1-SIRT2 axis.

## Discussion

Evidence across glucose, lipid, mitochondrial, immune, and autophagy related pathways indicates that SIRT1 and SIRT2 operate within an integrated regulatory system that supports tumor adaptation to metabolic and microenvironmental stress. Rather than functioning as simple antagonists, these two Sirtuins distribute metabolic control across distinct cellular compartments and regulatory levels, forming a Yin-Yang balance that coordinates biosynthesis, energy production, redox control, and immune modulation.

Within glucose metabolism, SIRT1 and SIRT2 bias metabolic flux toward different outcomes. SIRT1 predominantly reshapes glycolysis through transcriptional regulation and enzyme deacetylation that promote diversion of intermediates into biosynthetic pathways and metabolic plasticity. By contrast, SIRT2 more directly regulates cytosolic enzyme activity to control ATP generation and redox supportive pathways, including the pentose phosphate pathway. Together, these activities establish a dynamic range in which glycolytic flux can be redirected toward growth, stress adaptability, or energetic efficiency depending on nutrient availability and oxygen tension.

A comparable regulatory pattern is evident in lipid metabolism. SIRT1 primarily acts through nuclear and mitochondrial programs to promote fatty acid oxidation and metabolic adaptation under stress, while SIRT2 constrains tumor-associated lipogenesis by post-translationally regulating key cytosolic enzymes such as ACLY. These mechanisms govern acetyl-CoA partitioning and lipid fate decisions, limiting excessive lipid accumulation while preserving the capacity for rapid metabolic adjustment.

At the mitochondrial level, both Sirtuins converge on the PGC-1α axis but emphasize distinct aspects of mitochondrial control. SIRT1 enhances mitochondrial biogenesis and oxidative capacity, supporting the energy output required for metabolic adaptation, with context-dependent contributions to tumor progression or therapy resistance. SIRT2 maintains mitochondrial integrity by limiting oxidative stress and regulating mitochondrial protein acetylation. This coordinated regulation increases mitochondrial activity while maintaining redox balance, consistent with a Yin-Yang relationship between metabolic output and protection.

Autophagy represents a central integration node within this regulatory context. SIRT1 generally promotes autophagy initiation and flux to restore metabolic balance during nutrient stress, whereas SIRT2 modulates autophagic progression and intracellular trafficking, preventing excessive or prolonged catabolism. Through coordinated control, autophagy remains effective, enabling tumor cells to survive variable metabolic conditions.

Within the tumor immune microenvironment, the Yin-Yang balance becomes strongly cell type dependent. SIRT1 displays context-dependent immunomodulatory roles, supporting either immune suppression or antitumor immunity depending on tumor type, metabolic state, and immune composition. In contrast, SIRT2 functions more consistently as a metabolic checkpoint in CD8⁺ T cells, restraining glycolytic and mitochondrial capacity under tumor-associated metabolic stress. This distinction positions SIRT1 as a conditional immune regulator and SIRT2 as a constraint on effector T cell metabolism.

This integrated model has important therapeutic implications. Targeting SIRT1 or SIRT2 as single factors is unlikely to reflect the full complexity of their coordinated functions in cancer metabolism and immunity. Selective modulation of SIRT2 represents a promising strategy to enhance CD8⁺ T cell metabolic capacity and responsiveness to immune checkpoint blockade, particularly within metabolically restrictive tumor microenvironments. By contrast, therapeutic intervention targeting SIRT1 is expected to require precise biological stratification, given its context-dependent roles in tumor metabolism and immune regulation. In tumors characterized by SIRT1 driven glucolipid reprogramming, combined targeting of SIRT1 and key metabolic pathways may limit tumor metabolic plasticity while preserving antitumor immune activity.

Both Sirtuins play essential roles in genome maintenance, metabolic homeostasis, and immune regulation in normal tissues, as evidenced by multiple studies showing their involvement in physiological stress responses, redox balance, and energy regulation in diverse organs 5, 120, 126-130. Accordingly, broad or systemic inhibition increases the risk of impaired genomic stability, metabolic toxicity, and unintended immune perturbation. These considerations highlight the importance of cell type specific targeting, temporal control, and rational combination strategies that target tumor specific metabolic dependencies rather than global suppression of Sirtuin activity.

In summary, SIRT1 and SIRT2 form a Yin-Yang regulatory system that coordinates metabolic adaptation across biosynthesis, energy production, redox homeostasis, autophagy, and immune function. This balance enables tumors to survive under dynamic metabolic and immune pressures but simultaneously exposes vulnerabilities that can be therapeutically targeted. Viewing cancer metabolism through this integrated model provides a conceptual foundation for developing strategies that rebalance metabolic control rather than targeting individual pathways.

## Figures and Tables

**Table 1:** Distinct mechanisms and functions of SIRT1 and SIRT2 in glucose metabolism

Target/ Pathway	SIRT1: Mechanism & Function	SIRT2: Mechanism & Function
PKM2	Deacetylates Lys135/Lys206 → Dimer formation → Glycolysis ↑ →Diverts intermediates to biosynthesis ↑.	Deacetylates Lys305 → Tetramer formation → Glycolysis ↓ → ATP ↑, lactate ↓.
PGAM1/2	Deacetylates C terminus → Inhibits activity → Glycolysis ↓ → 3-PG production ↑.	Deacetylates Lys100 → Activates activity → Glycolysis ↑→ Redox capacity ↑.
G6PD	Deacetylates Lys403 → Enhances NADPH production indirectly → Supportive regulator.	Deacetylates Lys403 → Direct activation → NADPH ↑ → Redox homeostasis.
HIF-1α	Inhibits mTOR-HIF-1α axis → Glycolysis ↓; Non-tumor, destabilizes HIF-1α; Tumor, deacetylates Lys709 → Stabilizes HIF-1α; Deacetylates Lys674 → Transcriptional activity ↓ → PDK1 ↓.	Genetic loss, deacetylates Lys709 → Stabilizes HIF-1α; Inhibitors, destabilizes HIF-1α.
c-Myc	Cycloheximide assays, deacetylates Lys323 → Destabilizes c-Myc → Glycolytic transcription (*LDHA*) ↓. [35S]-methionine assays, stabilizes c-Myc.	Represses NEDD4 → Stabilizes c-Myc → Glycolysis ↑.

**Table 2:** Experimental systems supporting key SIRT1/SIRT2 metabolic functions

Key finding	Sirtuin	Experimental model	Perturbation type	Context & conditions	Evidence level (experimental system)
PKM2 deacetylation promotes anabolic glycolytic reprogramming (dimerization)	SIRT1	Neuronal cell lines; primary neurons	Transient overexpression or knockdown; site-specific mutants	Neurotoxic stress; Glucose replete, normoxia	In vitro (neuronal cell lines and primary neurons); In vivo (mouse Parkinson's disease model)
PKM2 deacetylation enhances ATP generating glycolysis (tetramerization)	SIRT2	Cancer cell lines; xenograft tumor models	Genetic knockout; rescue mutants	Tumor metabolic stress	In vivo (xenograft) + in vitro (tumor cells)
PGAM1 inhibition under glucose restriction	SIRT1	Cancer cell lines	Endogenous induction; nutrient stress	Low glucose	In vitro (cell lines)
PGAM2 activation via Lys100 deacetylation	SIRT2	Myoblasts; cancer cells	Site-specific mutants; genetic manipulation	Redox demand	In vitro (cells) + biochemical assays
HIF-1α suppression in immune cells	SIRT1	Conditional knockout mice	Cell type specific genetic deletion	Immune metabolism	In vivo (immune specific genetic models)
HIF-1α stabilization in tumor cells	SIRT1	Cancer cell lines	Genetic loss; pharmacologic inhibition	Hypoxia; NAD⁺ replete	In vitro (tumor cells)
HIF-1α suppression in tumor cells	SIRT2	Cancer cell lines	Genetic overexpression; pharmacologic inhibition	Hypoxia; tumor metabolic stress	In vitro (tumor cell models)
HIF-1α activation and survival signaling in neuronal cells	SIRT2	Neuronal cell lines	Pharmacologic inhibition	Hypoxia; neuronal stress	In vitro (neuronal cell models)
c-Myc transcriptional activation and growth signaling	SIRT1	Cancer cell lines; genetic mouse models	Genetic overexpression/loss; modulation of NAMPT-DBC1 axis	NAD⁺ dependent metabolic state	In vitro (tumor cells); in vivo (genetic models)
c-Myc protein stabilization and metabolic reprogramming	SIRT2	Cancer cell lines; xenograft tumor models	Genetic overexpression/knockdown; selective pharmacologic inhibition	Tumor metabolic stress	In vitro (tumor cells); in vivo (tumor models)
ACLY destabilization and suppression of lipid biosynthesis	SIRT2	Cancer cell lines; mouse tumor models	Genetic overexpression/loss; acetylation site mutants	Tumor metabolic stress	In vitro (tumor cells); in vivo (tumor models)
ACLY upregulation and fatty acid oxidation in renal fibrosis	SIRT1	Renal tubular epithelial cells; renal ischemia reperfusion models	Genetic modulation	Ischemia reperfusion injury; fibrotic stress	In vitro (cell models); in vivo (renal injury models)
FOXO1 driven lipolysis	SIRT1	Adipocytes; cancer cell lines	Genetic overexpression/loss	Energy metabolism	In vitro (cell models); in vivo (metabolic models)
FOXO1 mediated repression of adipogenesis	SIRT2	Preadipocytes; adipocyte differentiation models	Genetic overexpression/loss	Adipogenesis; differentiation programs	In vitro (adipocyte differentiation models)
PGC-1α activation and mitochondrial metabolic reprogramming	SIRT1	Cancer cell lines; mouse models	Genetic overexpression/loss; pharmacologic activation	Energy stress	In vitro (cell models); in vivo (genetic and disease models)

This table summarizes the primary experimental systems supporting each conclusion without assigning subjective strength rankings, allowing readers to independently assess robustness based on experimental context.

## Abbreviations

SIRT1: sirtuin 1
SIRT2: sirtuin 2
NAD^+^: nicotinamide adenine dinucleotide
PKM2: pyruvate kinase isozyme type M2
PGAMs: phosphoglycerate mutases
PGAM1: phosphoglycerate mutase 1
PGAM2: phosphoglycerate mutase 2
HIF-1α: hypoxia-inducible factor 1-alpha
HIF-1β: hypoxia-inducible factor 1-β
ATP: adenosine triphosphate
DNA: deoxyribonucleic acid
3-PG: 3-phosphoglycerate
2-PG: 2-phosphoglycerate
His11: histidine 11
PPP: pentose phosphate pathway
NADPH: nicotinamide adenine dinucleotide phosphate hydrogen
G6PD: glucose-6-phosphate dehydrogenase
G6P: glucose-6-phosphate
6PG: 6-phosphogluconate
SUMO1: small ubiquitin-like modifier 1
Ub: ubiquitination
GLUT1: glucose transporter type 1
HK2: hexokinase 2
LDHA: lactate dehydrogenase A
PDK1: pyruvate dehydrogenase kinase 1
MDSCs: myeloid-derived suppressor cells
mTOR: mechanistic target of rapamycin kinase
DCs: dendritic cells
Th9 cells: T helper type 9 cells
P300: E1A-binding protein p300
PHD2: prolyl hydroxylase domain 2
AK-1: Akt serine/threonine kinase 1
VHL: von hippel-lindau
ENO1: enolase 1
MEFs: mouse embryonic fibroblasts
NEDD4: neural precursor cell expressed, developmentally down-regulated protein 4
TM: thrombomodulin
PDHA1: pyruvate dehydrogenase E1 subunit alpha 1
TCA cycle: tricarboxylic acid cycle
FOXO: forkhead box O
PPARγ: peroxisome proliferator-activated receptor γ
SREBP1: sterol regulatory element-binding protein 1
ATGL: adipose triglyceride lipase
FASN: fatty acid synthase
AMPK: AMP activated protein kinase
LKB1: liver kinase B1
DBC1: deleted in breast cancer 1
HCC: hepatocellular carcinoma
PGC-1α: peroxisome proliferator-activated receptor gamma coactivator 1-alpha
ACLY: ATP-citrate lyase
SP1: transcription specificity protein 1
TFAM: mitochondrial transcription factor A
mtDNA: mitochondrial DNA
DLBCL: diffuse large B cell lymphoma
COX1: cytochrome c oxidase subunit 1
ND1: mitochondrial encoded NADH ubiquinone oxidoreductase core subunit 1
ND6: mitochondrial encoded NADH ubiquinone oxidoreductase core subunit 6
NSCLC: non-small cell lung cancer
NADH: nicotinamide adenine dinucleotide
UCP2: uncoupling protein 2
Bou: bouchardatine
DATS: diallyl trisulfide
ROS: reactive oxygen species
TME: tumor immune microenvironment
CX3CL1: CX3C motif chemokine ligand 1
SATB1: special AT-rich sequence-binding protein 1
BTG2: B cell translocation gene 2
IFN-γ: interferon-gamma
CXCL9: CXC motif chemokine ligand 9
CXCL10: CXC motif chemokine ligand 10
NF-κB: nuclear factor kappa-B
IL-12: interleukin-12
TGF-β: transforming growth factor beta
T-cell: T lymphocyte
FAO: fatty acid oxidation
